# Sclerosing melanocytic tumors with MAP2K1 in frame deletions and 15q gains: A distinctive pathway of nevogenesis with reproducible morphology

**DOI:** 10.1007/s00428-025-04368-z

**Published:** 2025-12-19

**Authors:** Ben J. Friedman, Olena Kis, Brandon Shaw, Jeffrey M. Cloutier, Shaofeng Yan, Joel A. Lefferts, Ahmed K. Alomari, Carina A. Dehner, Aofei Li, Andrea Moy, Konstantinos Linos

**Affiliations:** 1 Department of Dermatology, Henry Ford Hospital, 3031 West Grand Blvd, Suite 800, Detroit, MI 48202 USA; 2 Department of Pathology and Laboratory Medicine, Henry Ford Health, Detroit, MI USA; 3 Department of Pathology and Laboratory Medicine, Dartmouth-Hitchcock Medical Center, Lebanon, NH USA; 4 Laboratory for Clinical Genomics and Advanced Technology, Department of Pathology and Laboratory Medicine, Dartmouth Hitchcock Medical Center, Lebanon, NH USA; 5 Department of Pathology, Indiana University School of Medicine, Indianapolis, IN USA; 6 Department of Pathology, University of Pennsylvania, Philadelphia, PA USA; 7 Department of Pathology and Laboratory Medicine, Memorial Sloan Kettering Cancer Center (MSKCC), 1275 York Ave, New York, NY 10065 USA; 8 Michigan State University College of Human Medicine, Grand Rapids, USA

**Keywords:** MAP2K1, Melanocytic nevi, Chromosome 15 gain, Molecular dermatopathology

## Abstract

Previous work identified a subset of melanocytic nevi with distinctive histomorphologic features associated with gains of chromosome 15q. Subsequently, two additional cases with similar morphology demonstrated in-frame *MAP2K1* deletions together with 15q gains, suggesting a potential unifying genetic pathway. This study aimed to further characterize the molecular and clinicopathologic features of such lesions through retrospective and prospective analysis. A total of eleven melanocytic tumors were analyzed. This cohort included four cases from a previously published series as well as five newly identified, prospectively collected cases. For completeness, two additional tumors with known *MAP2K1* mutations and chromosome 15q gains—previously reported by some of our co-authors—were reanalyzed and incorporated into the overall analysis. All tumors underwent comprehensive histopathologic evaluation, assessment of 15q copy number status by SNP microarray, FISH, or whole exome sequencing, and next-generation sequencing to detect *MAP2K1* alterations and other relevant oncogenic events. All eleven tumors displayed reproducible histopathologic features: a dome-shaped silhouette, Clark’s/dysplastic nevus-like epidermal changes, and a prominent dermal component with sclerotic stroma. Each case harbored a copy number gain of chromosome 15q and an in-frame *MAP2K1* deletion. No lesion showed additional high-risk oncogenic mutations, aside from isolated findings in *TP53*, *NF1*, *FBXW7*, and *ROS1*. All *MAP2K1* variants were classified as Class 2 or Class 3, capable of activating the MAPK pathway independently of *BRAF* or *NRAS*. Follow-up data revealed indolent behavior, including one local recurrence without histologic progression. We describe a novel subset of melanocytic tumors defined by distinctive morphologic features, 15q copy number gains, and Class 2/3 *MAP2K1* in-frame deletions. These findings suggest a unique, pathway of nevogenesis. While biologically indolent, the presence of atypical features in select cases supports cautious clinical management, including complete excision and molecular correlation when appropriate.

**Supplementary Information:**

The online version contains supplementary material available at 10.1007/s00428-025-04368-z.

## Introduction

Previously, several of our co-authors (K.L., J.A.L., and S.Y.) published a series of eight melanocytic tumors with highly distinctive histomorphologic features, all demonstrating copy number gains of chromosome 15q [1]. All cases exhibited a dome-shaped silhouette with superficial features reminiscent of Clark’s/dysplastic nevi, including epidermal hyperplasia with bridging of adjacent rete ridges, lamellar fibroplasia of the papillary dermis, and lentiginous growth of pigmented melanocytes along the dermoepidermal junction. In contrast to typical Clark’s/dysplastic nevi, however, all lesions in the series showed a prominent broad and deep dermal component composed of variably sized epithelioid and spindled melanocytes embedded within a densely sclerotic stroma, with gradual dispersion of tumor cells toward the base.

The cytologic spectrum of the dermal melanocytes ranged from ovoid nuclei with inconspicuous nucleoli, and minimal cytoplasm, as seen in common nevi, to enlarged, epithelioid forms with prominent central nucleoli and abundant cytoplasm, reminiscent of Spitz. None of the 8 lesions expressed BRAFV600E or RAS Q61R by immunohistochemistry. SNP array analysis highlighted recurrent copy number gains on 15q, encompassing several oncogenes of interest, including *MYO5A*, *MAP2K1*, *SMAD3*, *PKM*, *PML*, *NTRK3*, *POLG*, and *IDH2*.

Following this initial report, 2 additional cases with nearly identical histopathologic features and 15q gains were identified by some of the co-authors (B.J.F., K.L. and A.A.). Notably, these cases also harbored in-frame deletions of *MAP2K1* [2, 3]. This observation prompted an inter-institutional collaboration to revisit the original cohort for targeted analysis of the *MAP2K1* locus and to further investigate the presence of additional pathogenic mutations in other cancer-associated genes. In parallel, five new cases meeting the same morphologic criteria were prospectively identified and included for confirmatory cytogenetic and molecular testing.

## Methods

### Selection of cases

Formalin-fixed, paraffin-embedded (FFPE) tissue blocks from Dartmouth Hitchcock Medical Center corresponding to previously reported melanocytic lesions with chromosome 15q gains were retrieved to assess suitability for next-generation sequencing (NGS) [1]. Of the original 8 cases, 4 had adequate material for further molecular testing. The two previously reported lesions with 15q gains and *MAP2K1* mutations were included as cases 5 and 6 in the current analysis [2, 3]. Additionally, 5 new cases with similar histomorphologic features were identified prospectively at collaborating institutions (Indiana University, Henry Ford Health, Memorial Sloan Kettering Cancer Center, and Dartmouth) and referred for cytogenetic and sequencing analysis [1–3]. The study was conducted with approval from the respective institutional review boards.

### Clinical data

Institutional electronic medical records from Memorial Sloan Kettering Cancer Center, Dartmouth Hitchcock Medical Center, Indiana University, and Henry Ford Health were queried for demographic and clinical information. Extracted data included patient age, gender, anatomic site, gross appearance, treatment details, and available follow-up.

### Histomorphologic features

All prospectively identified cases were reviewed independently by two board-certified dermatopathologists (B.J.F. and K.L.). Inclusion in the study was based on consensus agreement that the histologic features were consistent with the previously described entity.

### Copy number analysis for prospectively identified cases

Single-nucleotide polymorphism (SNP) microarray analysis was performed on formalin-fixed paraffin-embedded (FFPE) tissue using the OncoScan FFPE Assay, following the manufacturer’s protocol as previously described [4]. Data analysis and visualization were conducted using the Chromosome Analysis Suite Software and Variant Intelligence Applications™ (VIA) software from Bionano.

### Cytogenetic analysis based on next-generation sequencing

DNA next-generation sequencing (NGS) data from Tempus Labs Inc. were analyzed with CNVkit 31 in Python (v3.9) environments. Log2 values falling out of the interval between + 0.2 and − 0.25 were considered significant by the default algorithm. Genomic instability visualized on the scatter plot was interpreted and documented by a pathologist (AL) [5]. For case 11, copy number alterations were inferred from normalized read-depth and allelic imbalance data derived from DNA NGS data.

### Fluorescence in situ hybridization

One prospectively identified case (Case 8), which failed quality metrics for SNP array analysis, underwent FISH testing using a probe targeting 15q24 to assess for copy number gain.

### Next generation sequencing

#### Cases 1–4, 7, 10 and 11

DNA was extracted from formalin-fixed, paraffin-embedded tissue using the Ionic Purification System (Purigen Biosystems). Libraries were prepared using SureSelect Human All Exome v8 (Agilent) and sequenced on NovaSeq 6000 (Illumina). A custom clinically validated bioinformatics platform (AUGMET) was used for variant calling and interpretation [6]. Data were filtered using a set of 204 cancer-related genes (see Supplementary Table 3) and were reviewed for variants of potential biological relevance [6].

#### Cases 6 and 8

Tissue underwent testing with comprehensive next-generation sequencing (NGS) assay that targets 170 genes associated with solid tumors. Target libraries were prepared using Illumina TruSight Tumor 170 kit [7]. Sequencing was performed on a NextSeq Dx 550 instrument. SOPHiA DDM software was utilized for sequence alignment against reference genome, detection of sequence variants, fusions and CNVs, variant annotation and diagnostic, prognostic and therapeutic interpretation of analysis results.

#### Cases 5 and 9

Next-generation sequencing was conducted using the Tempus xT assay (Tempus Labs Inc.), as previously described [8, 9]. Briefly, Tempus xT is a targeted, tumor/normal-matched panel that detects single-nucleotide variants, insertions and/or deletions, and copy number variants in 648 genes, as well as chromosomal rearrangements in 22 genes [8, 9].

## Results

Clinical, pathologic, and molecular data from eleven melanocytic tumors considered to represent the same distinctive entity were aggregated (Table 1). Patient ages at the time of biopsy ranged from 36 to 83 years, with a mean and median age of 61. Three of the eleven cases occurred in male patients, while the remaining eight arose in females. All tumors originated on the trunk or extremities.

**Table 1:** Summary of melanocytic tumors with *MAP2K1* mutations and 15q gains

Case #	Age	Sex	Clinical Site	Immunohistochemistry	MAP2K1 mutation/allele frequency/depth of coverage	Additional Pathogenetic Mutations	Cytogenetic Findings	Treatment/Course	Follow Up
1 ^*^	59	Female	Right Upper Lateral Thigh	p16: retained expression	p.Ile103_Lys104del/25%/44	None	SNP array: Gain in 15q	Re-excision, clear margins	8 years, no recurrence
2 ^#^	40	Female	Left Upper Back/Infrascapular	None	p.Ile103_Lys104del/41%/78	*NF1* p.Arg416Ter (stop gain in exon 11 of 58, 10.26% VAD, only 4 reads *FBXW7* p.Arg222Ter (stop gain in exon 4 of 12, VAD 5.61%, 6 reads)	SNP array: Gain in 15q	Re-excision, clear margins	8 years, no recurrence
3 ^&^	60	Male	Central Mid Back	None	p.Gln56_Gly61delinsPro/17%/155	*ROS1* p.Trp133Ter (stop gain in exon 5 of 43, VAD 21.57%)	SNP array: Gain in 15q	Re-excision, clear margins	7 years, no recurrence
4 ^+^	74	Female	Left Upper Arm	None	p.Pro105_Ala106del/55%/590	None	SNP array: Gain in 15q and Loss of X	Positive margins; observation	5 years, no recurrence
5 ^	66	Female	Arm	SOX10: abundant pagetoid scatter p16: patchy weak expression, HMB-45: incomplete stratification	p.I103_K104del/16.8%/488	None	SNP array: Gain in15q and loss of X	Re-excision, clear margins and negative SLNBx	12 months, no recurrence
6^%^	61	Female	Right Lateral Leg	p16: focal loss PRAME: negative HMB45: patchy retained dermal staining	p.Q58_E62del/19.4%/1807	None	SNP array: Gain in 15q and Loss of X	Positive margins; recurred over 13 years	Re-excised with similar morphologic features evident, negative margins
7	78	Female	Left Upper Arm	p16: retained expression, PRAME: negative, HMB45: negative in dermis, Ki67: < 5%	p.Ile103_Lys104del/18%/59	*TP53* p.Gln331Ter (stop gain in exon 9 of 11, 5% VAD, 19 reads), *TP53* p.His179Asn (6.6% VAD, 37 reads)	SNP array: Gain in 15q	Re-excision, clear margins	9 months, no recurrence
8	83	Male	Left Calf	p16: retained expression, BRAFV600E: negative HMB45: negative	p.I103_K104del/21.5%/3403	None	FISH: Gain in 15q	Re-excision, clear margins	12 months, no recurrence
9	36	Male	Right Lateral Abdomen	p16: retained expression, PRAME: negative, Ki67 < 1%	p.P105_I107delinsT Inframe deletion – VAF 22.7%	None	15q gain **	Not re-excised, clinical follow up	15 months, no recurrence
10	51	Female	Left Posterior Upper arm	p16: retained expression, PRAME: 4 + (weak positive) MIB1: < 1%	p.Gln58_Glu62del/34%/277	None	SNP array: Gain in 15q and loss of 4p	Negative margins	23 months, no recurrence
11	65	Female	Right Elbow	p16: retained expression , PRAME: negative, Ki67: < 5%	p.Ile103_Lys104del/VAF 35%	None	15q gain **	Not re-excised	Recent case (no meaningful follow-up)

^*^ Case 2 in Olson et al*. 2021*

^*#*^* Case 3 Olson *et al*. 2021*

^*&*^* Case 6 Olson *et al*. 2021*

+ *Case 8 Olson *et al*. 2021*

^ *Case 4 Alomari *et al*. 2023*

^%^ *Case from Hamad *et al*. 2024*

^**^ Cytogenetic analysis based on next-generation sequencing

*FISH*, Fluorescence in situ hybridization; *SNP*, Single nucleotide polymorphism array

Four specimens from the original series by Olson et al. (2021) that had sufficient material for additional molecular testing were retrospectively found to harbor pathogenic in-frame deletions in *MAP2K1* (Cases 1–4, Table 1). Of these, two lacked additional pathogenic mutations in other known oncogenes, while one had additional mutations in *NF1* and *FBXW7* (Case 2) and the other had a *ROS1* mutation (Case 3). All four patients have been free of recurrence for 5 + years, including Case 4 which was not re-excised despite positive biopsy margins. Examination of all cases at low magnification revealed a dome-shaped exophytic configuration. Epidermal hyperplasia with bridging of adjacent rete pegs was present in the area of the lesion relative to the background epidermis. The dermal component was wedge-shaped and lesional cells showed sclerotic dispersion at the deep aspect. Varying amounts of melanin pigment was present in the background dermis.

**Fig. 1:**
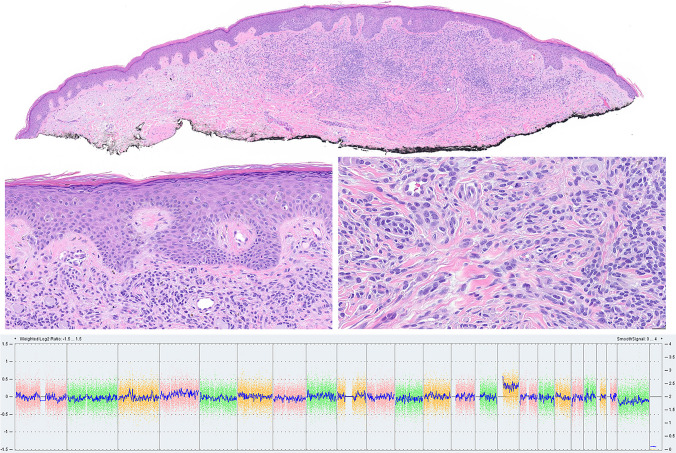
Case 7. Shave biopsy specimen from the left upper arm of a 78-year-old female (H&E, top panel). Greater pagetosis within the epidermal component and nuclear pleomorphism in the dermal component is identified in this case by comparison to the others. SNP array plot demonstrating isolated gain in 15q (bottom panel)

**Fig. 2:**
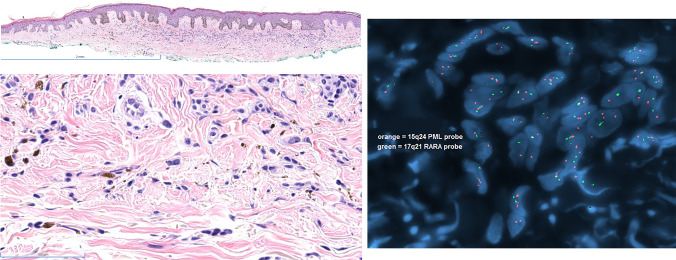
Case 8. Shave biopsy specimen from the left calf of an 83-year-old male (H&E, Left Panel). FISH image showing three copies of 15q24 (right panel)

**Fig. 3:**
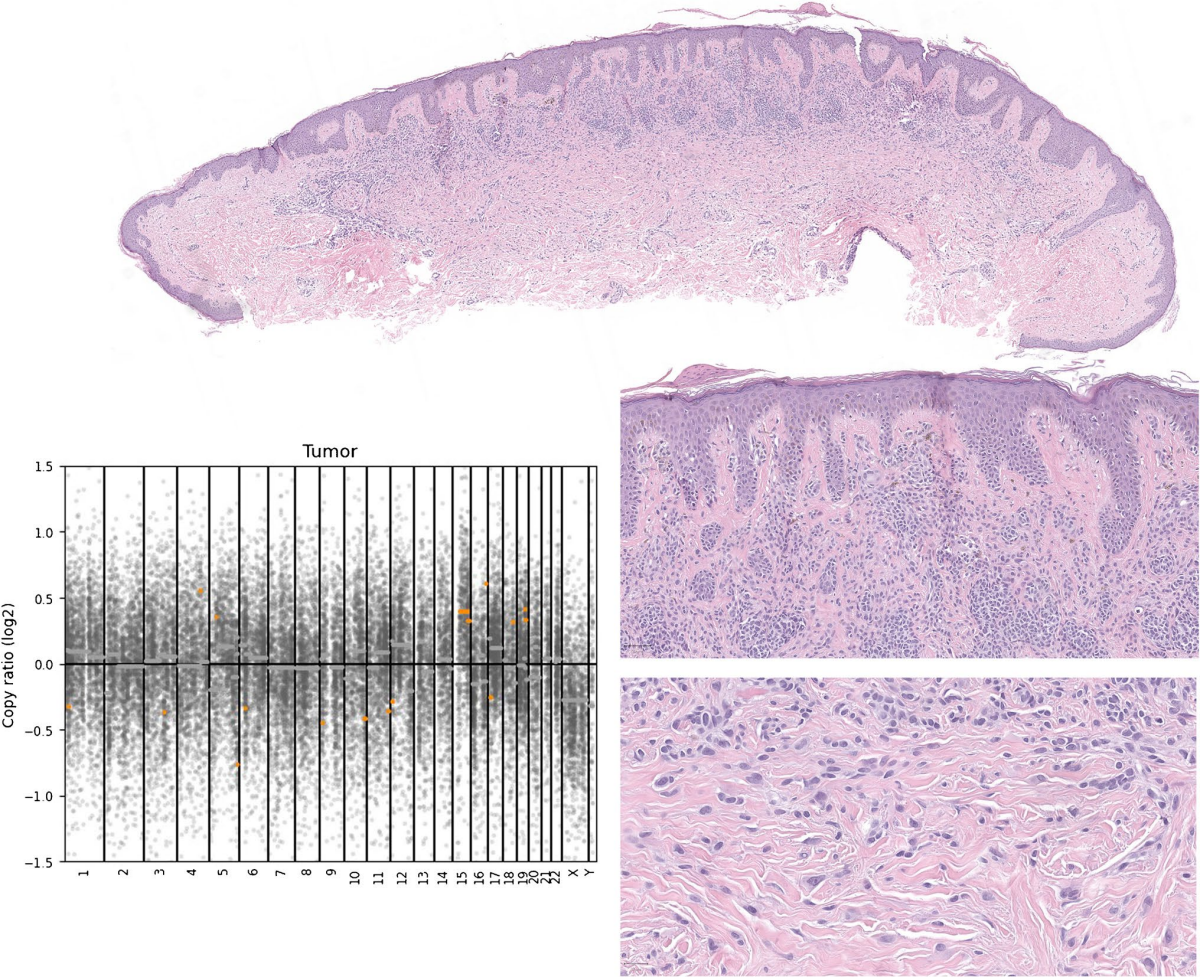
Case 9. Shave biopsy specimen from the right lateral abdomen of a 36-year-old male (H&E, Upper and Right Panels). Chr. 15 gain by CNV kit method (lower left panel)

**Fig. 4:**
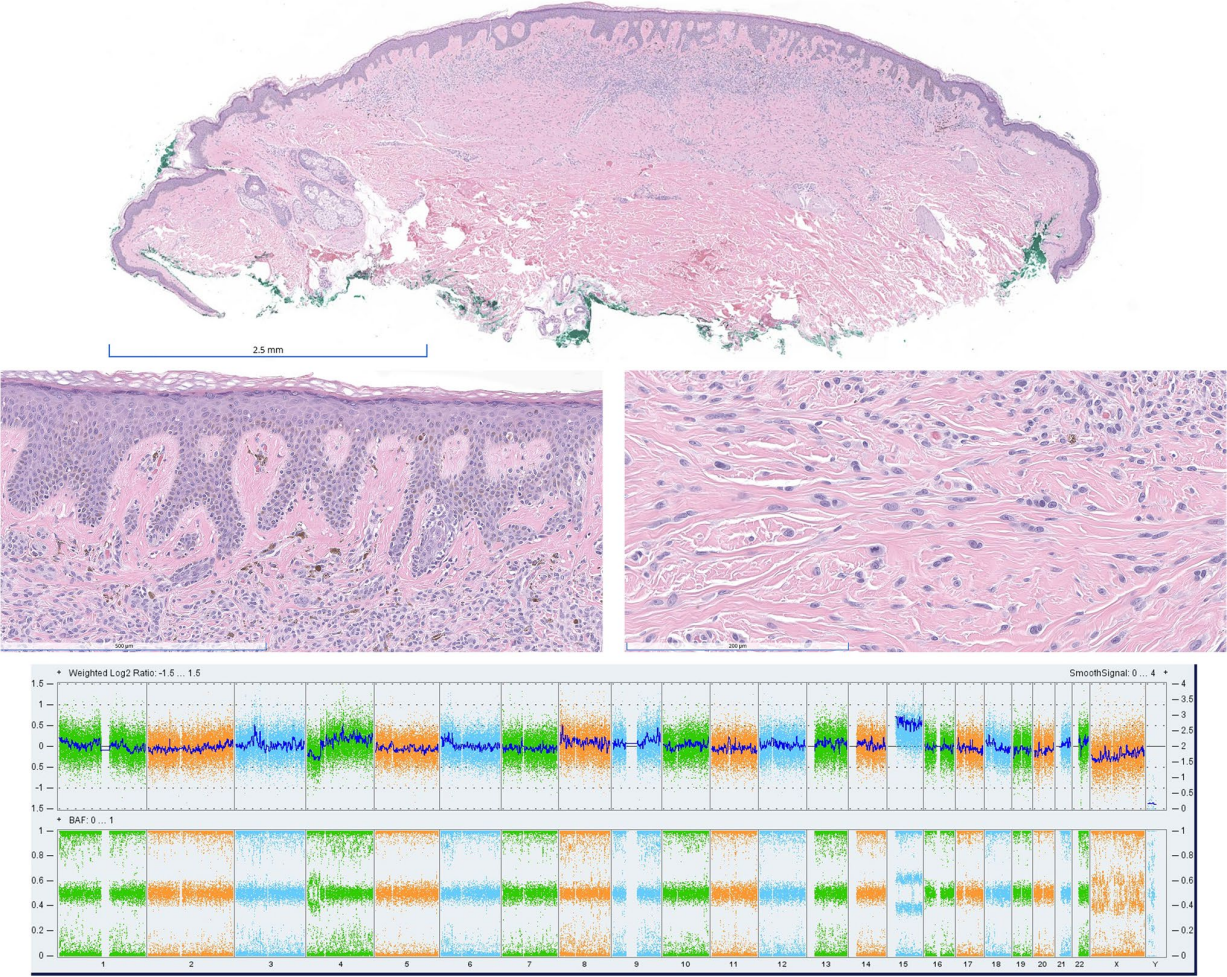
Case 10. Shave biopsy specimen from the left posterior upper arm of a 51-year-old female (H&E, top panel). SNP array plot demonstrating a gain in 15q and loss of 1 copy of 4p (bottom panel)

All five prospectively identified cases (Cases 7–11, Table 1) harbored 15q copy number gains and pathogenic *MAP2K1* in-frame deletions. None had additional pathogenetic mutations detected, except for case 7 which also demonstrated a mutation in *TP53* (Fig. 1). Cases 7 and 11 demonstrated the greatest architectural (pagetoid scatter) and cytomorphologic atypia within the cohort at large, though maintained the general pattern seen for Cases 1–4. Owing to the limited size and thin nature of the biopsy in case 8, SNP array analysis could not be performed. Instead, FISH confirmed the presence of a 15q gain (Fig. 2). On histomorphology, the dermal component in for Case 8 was the thinnest and least developed within the cohort, though maintained the epithelioid cytology and sclerotic background. In case 9, the isolated gain in 15q was detected via CNV kit method (Fig. 3). The histologic findings for case 9 was entirely consistent with the above-described pattern. Case 10 also demonstrated loss of 1 copy of 4p, a finding not seen in any of the other lesions. Nevertheless, the histomorphologic pattern remained identical (Fig. 4).

Two cases previously described in the literature by co-authors (B.J.F., K.L. and A.A)[Cases 5, 6, Table 1] were included and reviewed for completeness in this cohort given prior identification of *MAP2K1* in-frame deletions and gains in 15q in the context of near identical histomorphologic features of a dome shaped silhouette, epidermal hyperplasia with bridging of adjacent rete ridges, epithelioid cytology and dermal sclerosis [2, 3]. Both also demonstrated loss of 1 copy of chromosome X, a phenomenon felt to be age related. No patient in our cohort under the age of 61 demonstrated this finding. Neither Case 5 or 6 had any additional pathogenic mutations identified. Case 6 was the only tumor in this series to show a local recurrence, which underwent re-excision 13 years later with no evidence of histologic progression. All *MAP2K1* mutations across the entire cohort (Cases 1–11) fell into Class 2 or Class 3 categories, based on previously described functional subclasses: Class 1 (RAF-dependent), Class 2 (RAF-regulated), and Class 3 (RAF-independent) [Table 2] [10–12].

**Table 2:** *MAP2K1* mutations by variant class

Case	MAP2K1 mutation	Amino acid change	Variant class	Notes
1	p.Ile103_Lys104del	In-frame deletion	Class 3	RAF-independent, MEK-activating
2	p.Ile103_Lys104del	In-frame deletion	Class 3	Same as Case 1
3	p.Gln56_Gly61delinsPro	In-frame deletion	Class 2*	Regulatory domain alteration
4	p.Pro105_Ala106del	In-frame deletion	Class 3	Kinase domain alteration
5	p.Ile103_Lys104del	In-frame deletion	Class 3	Same as Case 1
6	p.Gln58_Glu62del	In-frame deletion	Class 2*	Regulatory domain alteration
7	p.Ile103_Lys104del	In-frame deletion	Class 3	Same as Case 1
8	p.Ile103_Lys104del	In-frame deletion	Class 3	Same as Case 1
9	p.Pro105_Ile107del	In-frame deletion	Class 3	Kinase domain alteration
10	p.Gln58_Glu62del	In-frame deletion	Class 2*	Regulatory domain alteration
11	p.Ile103_Lys104del	In-frame deletion	Class 3	Same as Case 1

^*^Predicted to have similar functional consequence to class 2 mutants based on structural similarity, but no direct evidence given rarity of variants

## Discussion

In recent years, the classification of melanocytic tumors has undergone a significant transformation, moving beyond traditional morphology to incorporate molecular and pathway-based frameworks. The 2022 WHO classification of melanocytic tumors outlines nine molecular classes, which are stratified based on oncogenic driver mutations, anatomic site, clinical context, and cumulative sun damage [13]. This biologic taxonomy captures the spectrum from benign nevi to intermediate grade “melanocytomas” to overt melanoma, allowing for more nuanced interpretation of ambiguous melanocytic lesions.

Within this framework, the mitogen-activated protein kinase (MAPK) pathway plays a central role in melanocytic tumorigenesis. One of its key components, *MAP2K1* (MEK1), encodes a serine/threonine kinase that lies downstream of RAF and upstream of ERK, mediating signal transduction from receptor tyrosine kinases and RAS proteins. Activating mutations in *MAP2K1* can lead to constitutive ERK signaling, driving melanocyte proliferation and survival. *MAP2K1* mutations have been reported across the full biologic continuum of melanocytic neoplasia [10]. In malignant melanoma, *MAP2K1* mutations, particularly in-frame deletions and complex indels, have been observed in approximately 2–8% of cases [14].

Several recent series have helped define the range of morphologies seen in benign and intermediate *MAP2K1*-mutated melanocytic tumors. Sunshine et al. described 6 cases with in-frame deletions in *MAP2K1*, showing a broad morphologic spectrum that included spitzoid cytomorphology, pigmented epithelioid melanocytoma (PEM)-like features, and deep penetrating nevus (DPN)-like plexiform architecture [15]. Donati et al. reported *MAP2K1*-mutated neoplasms with spitzoid cytology combined with architectural features of Clark/dysplastic nevi (“SPARK” nevi) [16]. Fumero-Velázquez et al. described 16 benign and intermediate-grade *MAP2K1*-mutated tumors classified as Spitz nevi, atypical Spitz tumors, or DPNs, noting a predominance of in-frame deletions in the less aggressive lesions [17]. In one large series, Ebbelaar et al. reported that 40/50 (80%) of *MAP2K1* mutated melanocytic nevi and melanocytomas had spitzoid histomorphology, with 11/27 (40%) of tested cases found to have a gain in 15q (no further defining characteristics discussed or photos shown) [10]. Collectively, these reports highlight that *MAP2K1*-mutated tumors encompass a variety of morphologies, most commonly spitzoid or DPN-like, but that architectural features of dysplastic nevi are also represented.

Our current series of 11 cases helps refine this spectrum by describing a more defined histologic pattern characterized by highly reproducible histomorphologic features including: a dome-shaped silhouette, Clark’s-like epidermal changes, dense dermal sclerosis, and a cytologic spectrum ranging from banal to mildly-atypical epithelioid melanocytes. Furthermore, all cases in the current series also demonstrated copy number gains of chromosome 15q — a finding also observed in the original eight cases and confirmed in all newly identified tumors [1]. Notably, the additional *MAP2K1*-mutated nevi in the present study phenotypically resembled all of the lesions in the original 15q gain cohort, although confirmatory genetic testing was only possible in 4 out of the 8 original cases. Whether additional deleterious co-mutations may alter this consistent histomorphologic pattern requires further investigation.

While the pathogenic significance of the 15q gain remains to be fully elucidated, its consistency across all ten cases suggests that it may represent a cooperating or even initiating event in this subset of melanocytic neoplasia. Notably, the *MAP2K1* gene resides at chromosome 15q22.31, raising the possibility that 15q gain could potentiate MAPK pathway activation through amplification of mutant *MAP2K1* alleles or increased expression of the wild-type gene. In other words, this gain—encompassing the *MAP2K1* locus—may contribute to tumorigenesis via increased gene dosage and consequent MAPK pathway activation.

Moreover, this chromosomal region includes several other genes of oncogenic or metabolic relevance, such as *SMAD3*, *NTRK3*, *PKM*, *IDH2*, and *PML*, any of which could theoretically contribute to melanocytic proliferation or stromal remodeling. While the functional role of these genes in melanocytic neoplasia remains unclear, their presence within the recurrently gained region warrants further investigation. Although speculative at this stage, the uniform presence of 15q gain across all *MAP2K1*-mutant lesions may point to a unique genomic context that supports or stabilizes this specific pathway of nevogenesis. This may be a similar phenomenon to other previously described activating mutations in melanocytic tumors (i.e. *HRAS* and *NRAS*) leading to segmental chromosomal gains that are often associated with distinctive histomorphologic features [18, 19]). Future functional studies will be necessary to explore the biological consequences of 15q gain in this setting. It is noteworthy that 7/26 melanomas also tested for copy number changes in Ebbelaar et al. demonstrated gains in 15q. This suggests that lesions with this chromosomal abnormality may have the potential for biologic progression to malignancy.

Interestingly, all eleven tumors harbored in-frame *MAP2K1* deletions, predicted to be classified as either Class 2 (RAF-regulated) or Class 3 (RAF-independent) mutations, consistent with prior structural–functional studies [6–8]. Mechanistically, Class 2 mutations result in constitutively active MEK1 proteins that remain RAF-regulated, while Class 3 mutations produce RAF-independent, autonomously active proteins. In contrast to Class 1 mutations, which are weak drivers and frequently occur with concurrent *BRAF* or *NRAS* mutations in melanoma, Class 2 and 3 mutations can act as sole drivers of tumorigenesis and are associated with a lower risk of progression, as demonstrated by Ebbelaar et al. [10]. Lack of representation of Class 1 mutations in our cohort is consistent with the indolent behavior observed, though follow-up time was limited. Moreover, the lack of secondary high-risk alterations such as *TERT* promoter or *CDKN2A* mutations further supports the interpretation of these lesions as biologically low-grade and on the benign-to-intermediate end of the melanocytic neoplasm spectrum.

Despite their generally bland appearance, some tumors in our cohort displayed concerning features. Specifically, Cases 5, 6, 7, and 11 showed pagetoid spread, focal cytologic atypia, and in one instance, recurrence after incomplete excision. Interestingly, Cases 5, 7 and 11 all had Class 3 *MAP2K1* mutations which at least raises the possibility that Class 3 may confer slightly greater risk than Class 2. However, none of these lesions progressed to overt malignancy during follow-up (ranging up to 8 years). Whether these findings reflect early malignant progression or represent outliers within an otherwise indolent group remains unknown. Given these uncertainties, we advocate for complete excision of *MAP2K1*-mutant nevi that show significant atypia, pagetosis, or impaired maturation—paralleling our approach for atypical *BRAF*-mutant nevi. This cautious stance reflects both the known oncogenic potential of Class 2 and 3 *MAP2K1* mutations and the occasional recurrence seen in our series.

In summary, we have described a series of 11 low-grade melanocytic tumors with reproducible histopathologic features that harbor Class 2 and 3 *MAP2K1* in-frame deletions and copy number gains in 15q. Although none of the lesions demonstrated adverse behavior and they are suspected to represent nevi, some showed cytologic atypia, with one case developing a local recurrence. As our understanding of *MAP2K1*-mutated neoplasia evolves, particularly in the context of low-grade or borderline melanocytic lesions, integration of molecular findings with morphologic assessment can be helpful in guiding management.

## Supplementary information

Below is the link to the electronic supplementary material.

Supplementary file1 (DOCX 27.2 KB)

## Data Availability

Data is available upon reasonable request.
