# Supplementary material for: Sclerosing melanocytic tumors with MAP2K1 in frame deletions and 15q gains: A distinctive pathway of nevogenesis with reproducible morphology
Source: Virchows Arch. 2025 Dec 19;489(1):189–98. doi: 10.1007/s00428-025-04368-z (PMC13368881; doi:10.1007/s00428-025-04368-z)
Supplement: Supplementary file 1 — Supplementary file1 (DOCX 27.2 KB) [file 428_2025_4368_MOESM1_ESM.docx]

**Supplementary Table 3.** Cancer-related genes analyzed by DNA sequencing:

H3˗3A,ABRAXAS1,APC,AR,ARID1A,ARID1B,AKT1,AKT2,AKT3,ALK,ATM,ATR,ATRX,BAP1,BARD1,BCL2,BCL6,BRAF,CARD11,CBL,BRCA1,BRCA2,BRIP1,BTK,CCND1,CCND2,CCND3,CCNE1,CD79A,CD79B,CDH1,CDK12,CDK4,CDK6,CDKN1B,CDKN2A,CDKN2B,CDKN2C,CEBPA,CHEK1,CHEK2,CREBBP,CSF1R,CSF3R,CTNNB1,DDR2,EGFR,EP300,EPCAM,ERBB2,ERBB3,ERBB4,ERCC1,ERCC2,ERG,ESR1,ETV6,DNMT3A,EZH2,FOXL2,FANCI,FANCL,FBXW7,FGF1,FGF10,FGF14,FGF19,FGF2,FGF23,FGF3,FGF4,FGF5,FGF6,FGF7,FGF8,FGF9,FGFR1,FGFR2,FGFR3,FGFR4,FH,FLT1,FLT3,HNF1A,GATA1,GATA2,GATA3,GEN1,GNA11,GNAQ,GNAS,HRAS,JAK2,JAK3,KDR,IDH1,IDH2,KIT,KMT2A,KRAS,LAMP1,INPP4B,MAP2K1,MAP2K2,MAP3K1,MAP3K13,MAPK1,MCL1,MDM2,MDM4,MED12,MET,MLH1,MLH3,MLLT3,MPL,MRE11,MSH2,MSH3,MSH6,MTOR,MUTYH,MYC,MYCL,MYCN,MYD88,NBN,NPM1,NRAS,NRG1,NF1,NF2,NOTCH1,NOTCH2,NOTCH3,PDGFRA,PDGFRB,PIK3CA,PIK3CB,PIK3CD,PIK3CG,PIK3R1,PALB2,PTCH1,PTEN,PTPN11,PMS2,POLD1,POLE,PPP2R2A,RAD51,RAD51B,RAD51C,RAD51D,RAD54L,RAF1,RB1,RET,RICTOR,ROS1,RPS6KB1,RUNX1,SDHB,SDHD,SETBP1,SETD2,SLX4,SMAD4,SMARCA4,SMARCB1,SMO,SRC,STK11,SUFU,TERT,TET2,TFRC,TP53,TRAF7,TSC1,TSC2,VHL,XRCC2,YAP1,ACVR1,ZFHX3,DAXX,FAT1,FANCA,NFE2L2,PIM1,PTPRD,WT1,XPC,XPO1,WRN,PPM1D,KEAP1,PTPRT,DICER1,CD274,PBRM1,KMT2C,ARID2
